# Ecotoxicity of Recycled Aggregates: Application of a Prediction Methodology

**DOI:** 10.3390/ma15103510

**Published:** 2022-05-13

**Authors:** Margarida B. Maia, Jorge de Brito, Isabel M. Martins, José D. Silvestre

**Affiliations:** 1 Civil Engineering Research and Innovation for Sustainability (CERIS), Department of Civil Engineering, Architecture and Georresources, Instituto Superior Técnico, University of Lisbon, 1049-001 Lisbon, Portugal; anambraga@tecnico.ulisboa.pt (M.B.M.); jose.silvestre@tecnico.ulisboa.pt (J.D.S.); 2 Department of Materials, Laboratório Nacional de Engenharia Civil (LNEC), Av. do Brasil 101, 1700-066 Lisbon, Portugal; imartins@lnec.pt

**Keywords:** recycled aggregates, ecotoxicity, prediction methodology, sustainable construction

## Abstract

Due to environmental concerns, the search for sustainable construction solutions has been increasing over the years. This global concern is creating a trend in the use of recycled aggregates resulting from construction and demolition wastes from different sources. In addition to their physical and mechanical properties, it is important to analyse their ecotoxicological risk to determine whether their leachates might be an issue. To assess ecotoxicity, biological tests should be performed for different trophic levels. This type of test is expensive and needs a high level of expertise, which leads to a lack of studies on recycled aggregates including ecotoxicity analysis. This paper presents a set of predictive ecotoxicity results based on the published studies on recycled aggregates. These results are the outcome of applying an innovative methodology previously developed and validated by the authors aiming to foresee the ecotoxicological fate of building materials’ constituents and products. The application of this methodology enables the classification of a recycled aggregate product as safe or unsafe in terms of ecotoxicity risk, while keeping biological testing to a minimum.

## 1. Introduction

Construction and demolition waste (CDW) represented a ratio higher than 35% of all waste generated in the EU in 2018 [1]. Taking into consideration these numbers, one of the EU waste policy objectives is to recover high-quality resources from CDW as much as possible to contribute to the circular economy. This concern has led not only to the increased incorporation of CDW as cement substitutes [2,3] but also to the use of several types of recycled aggregates (RAs) in road pavements [4,5], cementitious mortars [6,7] and concrete [8,9].

Over the last few years, several studies related to RAs’ physical and mechanical properties were developed. However, their potential ecotoxicological risk in living organisms is rarely assessed, and it cannot be disregarded due to the high heterogeneity of RAs and eventual exposure to components containing dangerous substances [10,11]. RAs’ leachate can interact with the ecosystem, and its toxicity can affect all the surrounding elements. Thus, determining whether these eluates are toxic to the aquatic environment is essential [12,13].

Ecotoxicity, defined by the study of the toxic effects on ecosystems caused by natural or synthetic pollutants, corresponds to one of the materials’ hazardous properties, HP 14 [14]. Basically, in order to estimate ecotoxicity, it is possible to apply chemical analyses or biological tests. CEN/TR 17105:2017 [15] is a technical report that presents the biological approach to evaluate construction products’ ecotoxicity and states that chemical analyses may not be the most appropriate means of estimation of toxicity for individual substances for complex products of unknown composition.

For the application of biological toxicity tests in this scope, it is required to expose organisms from a minimum of three different trophic levels to the diluted eluate resulting from the leaching of RAs. The aquatic ecotoxicity expressed as the effective concentration of a substance (EC50) that causes 50% of the maximum response can be obtained, as an example, through Luminescent bacteria [16] (ISO 11348-3), Algae [17] (ISO 8692) and *Daphnia magna*, the last representing Crustacean [18] (ISO 6341).

There are already several studies that analyse the chemical composition (CC) of RAs’ eluate according to different aspects (e.g., pH, L/S, age, etc.) [19,20,21]. Some of these studies conclude that chromium and sulphate are amongst the most critical components [11,22,23,24]. It is possible to compare the content of analytes released from RAs, based on their concentration in the eluate, with legal limit values, allowing for example their classification for landfill disposal for inert, non-hazardous or hazardous waste [11,25,26]. By contrast, there are not many studies including biological tests of RAs’ eluate.

Römbke [27] used species from three different trophic levels as aquatic test species, concluding that mixed construction waste presented no toxicity concern level. By testing species from three different trophic levels, Lalonde et al. [28] applied ecotoxicity tests to several materials, including concrete, and ranked their toxicity level, which is expressed by lethal concentration, LC50.

Brás et al. [29] and Choi et al. [30] claim that the incorporation of toxic raw materials in concrete results in environmental benefits. The first study, by evaluating the toxicity effects in terms of the growth of duckweed fronds, indicates that concrete incorporating fly ashes from a thermoelectric plant and lime sludge from a paper mill is safer than a reference concrete [29]. Likewise, concrete prepared with wastes (e.g., pulverised fuel ash, pozzolanic admixtures, ground granulated blast furnace slag with or without loess) presented lower ecotoxicity than eluates from each corresponding waste, according to Choi et al. [30], who made tests with *daphnia magna*.

Rodrigues et al. [25] and Mocová et al. [31] investigated the ecotoxicity of conventional concrete and recycled concrete aggregate (RCA), concluding that these materials’ eluates are toxic for the aquatic environment. These results were obtained using the original leachates (without any treatment). Thus, is not possible to determine whether the toxic effect is due to the highly alkaline pH of concrete or to other factors [31]. Rodrigues et al. [25], who also analysed the toxicity of each raw material, concluded that the toxicity of concrete with fly ash is lower than the one of fly ash.

Four fine RAs were studied by Mariaková et al. [32], and half of them, which present higher pH levels, were classified as toxic. The authors also claim that the use of waste materials in concrete compositions leads to the immobilisation of toxic elements.

In an attempt to minimise laboratory tests, Rodrigues et al. [33] proposed a methodology for the ecotoxicological characterisation of virgin and recycled raw materials and construction materials, allowing the classification of raw materials (virgin or processed) without determining the CC and performing an ecotoxicological characterisation of the leachates. Nevertheless, in the case of raw materials that are recycled or sub-products, and of construction materials, biological tests and CC are still needed.

This type of biological test is expensive and needs a highly specific knowledge level, which leads to a lack of RA studies that include ecotoxicity analysis. Therefore, an innovative methodology previously developed and validated by the authors [34] was applied to previously published studies involving 51 RA samples. These studies present different types of information about RAs to simulate different types of research paths.

This paper presents a set of ecotoxicity predictive results determined by applying this innovative methodology that foresees ecotoxicological fate. Its application enables the classification of a RA product as safe or unsafe, in terms of ecotoxicity risk, reducing the need for CC and biological tests.

## 2. Materials and Method

### 2.1. Materials

Sixty samples of RAs were selected from previous studies. Table 1 briefly presents the samples considered. There was special care to select studies that analysed different RA properties, e.g., their CC or of their eluate. Different methods were used by the authors to define RA properties, which are presented in Table 2. In Table A1 and Table A2 of Appendix A, the detailed results obtained by the different authors related to the metal contents, anion content and dissolved organic carbon (DOC) of RAs’ eluate (Table A1) can be found.

### 2.2. Method

A new methodology, created by the authors to estimate whether a composition of a cement-based product (CBP) might have a worrying toxicity level, was summarily presented in a previous study [34]. By minimising the number of needed toxicity tests, this methodology intends to help researchers to increase the efficiency of resources. It is important to state that it is suggested to perform toxicity tests according to the corresponding standards, in case no information is available or of any suspicion that some product may have a toxicity level of concern. Since RAs are one CBP constituent, it is possible to apply this methodology to estimate their toxicity. In this particular case, RA was considered a product, not a constituent.

**Figure 1:**
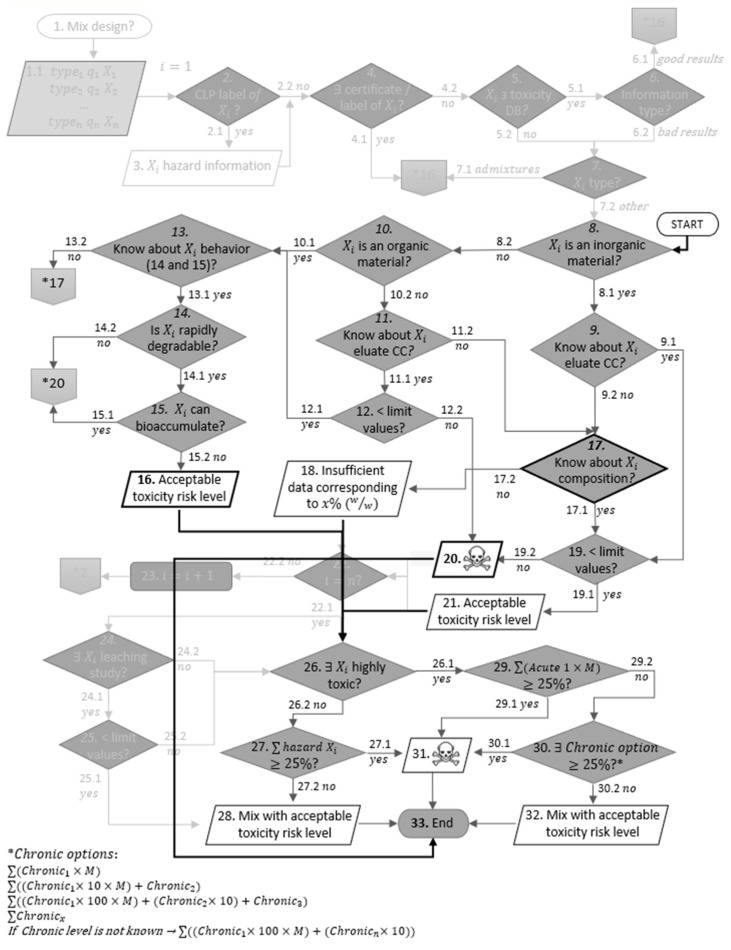
Flowchart of the applied methodology (adapted from [34]—original flowchart as watermark and adaptations highlighted in bold).

Figure 1 and Table A2 of Appendix B describe the methodology to be applied.

RAs have a high heterogeneity, and their exact constituents are not known; thus, there is no environmental label, certificate or database that includes the considered RAs’ ecotoxicity. With this in mind, the methodology was applied from step 8, i.e., from the step where the methodology assumes the division between organic and inorganic materials. The chemical characterisation of the eluate of the inorganic material, or its behaviour in the environment, should be studied. Conservatively, if there is no information available about the eluate’s CC resulting from a leaching test, then the CC of the material itself should be considered. The results from the CC of the eluate should be compared with the legal limits for waste acceptable in landfills for inert waste defined in national or European Union laws (Table 3) [42]. When analysing an organic material, it is not acceptable to exclude the risk of toxicity hazard if the compound can bioaccumulate and is not rapidly degradable. In case of doubt, RAs shall be assessed as both organic and inorganic material. Step 22, which would allow restarting the flowchart for the next component will not be considered, since RA is admitted as a singular product. For the same reason, steps 24 and 25, which are equivalent to steps 11 and 12, were not considered. Taking into account that this methodology was created for CBP and RAs are analysed as a product, whenever its application reaches step 20, the next step should be step 33. The end output is a list where RAs can be assigned to one of three options: “acceptable toxicity level”, “insufficient data”, or “material may have a toxicity concern level”. This classification list is obtained from the application of a variation of the “summation method” specified in Classification, Labelling and Packaging (CLP) regulation [43].

It is possible to find more information related to this methodology in the previous study of the authors [34].

## 3. Results and Discussion

When applying this methodology, the following assumptions have been considered:

- Only studies applying leaching tests that allow the application of limit values of Council Decision 2003/33/EC [42] were selected;
- Since RAs may have been exposed to organic contaminants, they should be considered both as organic and inorganic materials;
- Limit values of concentrations were assumed in case there is no information on a specific value (25% of the total analysed RAs);
- A quantification limit value was assumed in the cases in which they are below it (20% of the total analysed RAs);
- All components compounds were considered when there are no available compound data (just one RA);
- The compound was assumed as non-rapidly degradable if there is no information about it or other necessary values (e.g., BOD5, COD, $O_{2}{\mathrm{depletion}}_{28\;days}$, $CO_{2}{\mathrm{production}}_{28\;days}$);
- In the case of duplicated data from different sources, the worst-case scenario was admitted;
- All components were assumed in the form declared by the authors.

All 51 samples were analysed using the presented methodology, and Table A3 of Appendix C shows the methodology results step-by-step for each RA type.

**Figure 2:**
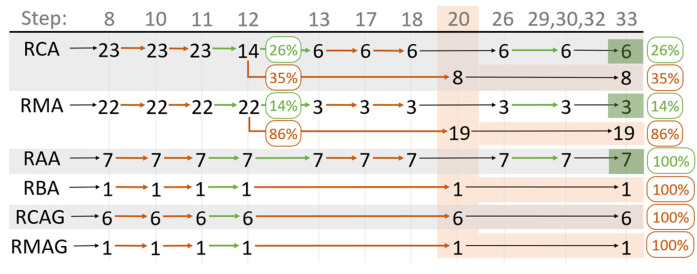
Quantitative methodology application flow by RA type (each cell represents the quantity of RA in each step of the methodology, orange arrows correspond to option “no”, green arrows correspond to option “yes”, orange cells correspond to “material may represent a toxicity concern level” and green cells correspond to “acceptable toxicity risk level”).

Only 31% of analysed RAs are not expected to have a toxicity concern level. Assessing by type of RA, the corresponding numbers are 26% for RCA, 14% for RMA, 100% for RAA and 0% for the remaining materials, as can be seen in Figure 2. Considering only the RA that does not have a leaching study, it is estimated that all of them have a toxicity concern level based on their composition. This is because the methodology became significantly more conservative when only RA CC is considered, since the release mechanisms in leaching tests, such as solubility and adsorption, do not relate to the total content of contaminants [22].

**Figure A1:**
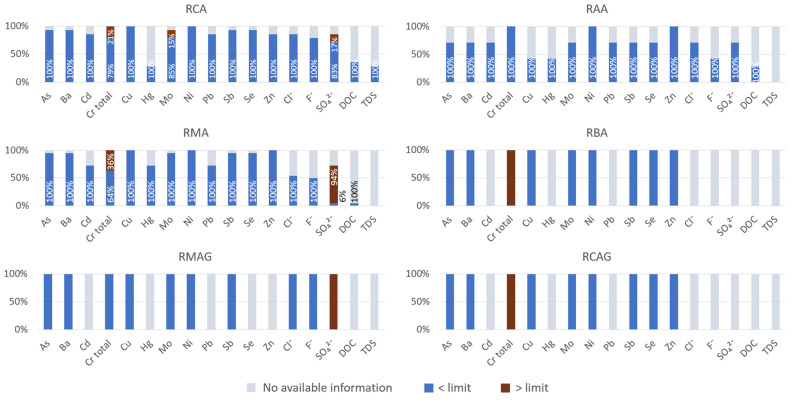
Percentage of RA whose eluate complies or not with the content limits under European Union legislation [41] for each analyte by type of RA (the percentage in each column considers only available information) References.

Reviewing step 12 that checks whether leached concentrations lead to released contents under the legal limit values, and in accordance with Figure 1, it is verifiable that chromium and sulphate exceed the limits by the following percentages, respectively: 21% and 17% for RCA, 36% and 94% for RMA, and 0% in both contents for RAA. In the case of RAs with gypsum contamination, all RCAG present Cr above limit values (but no values over the threshold for sulphate) and RMAG present SO_4_^2−^ above the limits. Molybdenum content released is critical for 15% of RCA. Please note that all these percentages (presented in Figure A1 of Appendix D) correspond to the average values of the RA studies that present information about that particular analyte. On the other hand, from those percentages that present metal and anion contents under the limits, only five RAs presented dissolved organic carbon (DOC) values above the limit.

Only 31% of the studied RAs reached step 21 as having an acceptable toxicity risk level, corresponding to six RCAs, three RMAs and seven RAAs. It should be noted that Barbudo et al. [23], the author of 44% of the referred RAs and all three RMAs, did not check the sulphate content released, which is one of the RMAs’ critical analytes, as pointed out by different authors [11,22,23]. The remaining 56% are all collected directly from treatment plants. From these, 67% correspond to crushed materials from a single material (100% of crushed concrete or bituminous pavement). None of these RAs presented any expression from steps 27, 29 or 30 higher than 25%, ending without having an estimated toxicity concern level according to this methodology.

Among the selected studies, only Rodrigues et al. [25] performed biological toxicity tests. Their results showed some toxicity level in all selected RAs, as well as when applying this methodology.

The next subsections detail intermediate calculations and validations required to apply this methodology.

### 3.1. Classification by Environmental Hazard Category

In step 26 of the methodology, a definition of the environmental hazard category of all RAs or their eluate components is required.

Table 4 presents the hazard list of all needed components [44] to apply the methodology to the selected RAs. Those not detailed in Table 4 were considered safe in terms of short- and long-term environmental hazards. All these data were collected from the PubChem site [44] that presents a large collection of chemical information, including European Chemicals Agency (ECHA) [45] and CLP information [43].

Components classified as environmental hazard category 1 (Acute 1 or Chronic 1) are classified as highly toxic and highlighted in bold in Table 4.

### 3.2. M-Factors for Highly Toxic Components

To check the inequalities established in steps 29 and 30, the definition of the appropriate multiplying factor (M factor) of highly toxic components is required. This M factor is defined taking into account the toxicity value for each component, as detailed in CLP regulation [43] and summarized in Table 5.

Since there is no available information about L(E)C_50_ or NOEC on the selected studies, these data were collected from the available online databases. The Ecotox database [46] provides information about chemicals and their effects on aquatic and terrestrial species. To define each M factor, all available studies from this database that present the required data were selected.

For example, for Cadmium, 34 studies that analysed LC_50_ for 96 h in standard fish species were selected. The value of 0.11 mg/L presented in Table 6 represents the weighted average values of the selected studies. The same procedure was followed for each presented concentration. All concentrations and M values are listed in Table 6. The lowest concentration of the fish, crustacean or algae studies (highlighted in bold) was the one considered for M acute value definition. It shall be noted that according to CLP regulation [43], for hazard categories, the definition of a specific test duration for each species should be considered.

For the M factor definition, some assumptions have been considered:

- Minimum concentration values were considered when the average value was not available;
- Only studies involving standard species were considered;
- Values above one were not considered, since they were considered as outliers, except in the case of LC_50_ fish (As), LC_50_ crustacea (Pb), NOEC (Pb) (values above 20 were also excluded) and LC_50_ fish or crustacean (Zn) (values above three were also excluded);
- The M acute factor of arsenic (As) was considered equal to one, since available values are all above one.

### 3.3. Calculations for RAs’ Hazard Level Definition

In steps 29 and 30, a classification of RAs based on the summation method details at CLP regulation [43] was defined. If the sum of the concentrations in percentage of RAs’ components multiplied by their respective M factors is higher than 25%, then the RA type shall be classified as Acute 1. Likewise, if any expression presented in Table 7 is higher than 25%, the RA type shall have the corresponding classification and a toxicity concern level. Table 7 presents all calculations for individual RA types. This information enabled finding that none of the RA types presented a result higher than 25% in all inequalities. Thus, it is not expected that any of the RA types considered in steps 29 and 30 present a toxicity concern level.

## 4. Conclusions

A methodology developed by the authors was applied to 51 RA samples to predict their ecotoxicity. Only 16 RAs concluded the methodology without an estimation of a toxicity concern level, and it is not therefore necessary to apply biological tests. Among these, only for nine RAs all RAs’ critical released contents indicated in previous studies (Cr and SO_4_^2−^) were evaluated. It is important however to note that several authors from the selected RAs did not analyse all released contents limits under European Union legislation [42], having admitted lower values than the legal limit content.

For the remaining RAs for which it was not possible to predict a toxicity concern level or present an estimation of a toxicity concern level, more information should be obtained or biological tests should be applied to estimate or obtain the toxicity concern level of each one.

Taking into consideration the results obtained through the application of the methodology to the selected RA, the conclusions are drawn as follows:

- It is more appropriate to apply this methodology using eluate’s information rather than RA composition;
- When analysing the RAs’ toxicity potential using this methodology, leaching limit values are more restrictive than the summation method from CLP regulation [43];
- Chromium and sulphate-released contents are RAs’ critical analytes, although only the first one is a highly toxic component;
- The studied RMA showed higher critical analyte released contents, increasing their toxicity potential;
- It is estimated that RAs composed of 100% of crushed concrete or 100% asphalt pavement present lower toxicity levels, not corresponding to a toxicity concern level (considering only these 51 RAs).

It is important to mention that the method is pending a validation, which is currently being completed.

## Figures and Tables

**Table 1:** RA types in the studies considered.

#	RA Type	Reference	
1–7	RCA	C1–C7	[22]
8–17	RMA	M1–M10	[22]
18–20	RAA	A1–A3	[22]
21–22	RCA	C1–C2	[23]
23–24	RAA	A1–A2	[23]
25–30	RMA	M1–M6	[23]
31	RBA	B	[23]
32–37	RCAG	X1–X6	[23]
38	RMAG	MRG1	[11]
39	RMA	MRW2	[11]
40	RCA	RA, A1–A3	[25]
44–47	RMA	MRA-A-D	[35]
48	RCA	A	[26]
49–50	RAA	E-MB, EP	[26]
51	RMA	T	[26]

RCA—recycled concrete aggregate, RMA—recycled mix aggregate, RAA—recycled asphalt aggregate, RBA—recycled crushed brick aggregate, RCAG—recycled concrete aggregate with gypsum contamination, RMAG—recycled mix aggregate with gypsum contamination.

**Table 2:** Methods/standards used in the studies selected.

	Methods/Standards
Leaching tests	EN 12457-3 [36], EN 12457-4 [37]
Chemical composition	XRF
Minerals	XRD
Heavy metals	ICP-MS, ICP-OES, GFAAS
Ions	Ion Chromatography (EN ISO 10304-1 [38])
Total dissolved solids, TDS	SMEWW 2540 [39]
DOC	SMEWW 5310 [40], LNEC E 386 [41]

XRF—X-ray fluorescence, XRD—X-ray diffraction, ICP-MS—Inductively coupled plasma mass spectrometry, ICP-OES—Inductively coupled plasma optical emission spectrometry, GFAAS—Graphite furnace atomic absorption spectroscopy, SMEWW—Standard methods for the examination of water and wastewater, LNEC E—Technical specification of Laboratório Nacional de Engenharia Civil.

**Table 3:** Leaching limit values for inert waste acceptable at landfills [42].

Component	L/S = 2 L/kg	L/S = 10 L/kg
	mg/kg Dry Substance	mg/kg Dry Substance
As	0.1	0.5
Ba	7	20
Cd	0.03	0.04
Cr total	0.2	0.5
Cu	0.9	2
Hg	0.003	0.01
Mo	0.3	0.5
Ni	0.2	0.4
Pb	0.2	0.5
Sb	0.02	0.06
Se	0.06	0.1
Zn	2	4
Chloride	550	800
Fluoride	4	10
Sulphate	560	1000
DOC	240	500
TDS	2500	4000

**Table 4:** Classification of components by acute and chronic hazards [44].

Name	CAS n.	Ideal Formula	Acute Level	Chronic Level	Reference
Iron(III) oxide	1309-37-1	Fe_2_O_3_		2	[45]
Sulphur trioxide	7446-11-9	SO_3_		2	[45]
**Arsenic**	7440-38-2	As	1	1	[43,45]
**Cadmium**	7440-43-9	Cd	1	1	[43,45]
**Chromium**	7440-47-3	Cr total	1	1	[45]
**Copper**	7440-50-8	Cu	1	1	[45]
**Mercury**	7439-97-6	Hg	1	1	[43,45]
Nickel	7440-02-0	Ni		3	[45]
**Lead**	7439-92-1	Pb	1	1	[45]
Antimony	7440-36-0	Sb		2	[45]
Selenium	7782-49-2	Se		4	[43,45]
**Zinc**	7440-66-6	Zn	1	1	[43,45]

**Table 5:** M factors for highly toxic components [43].

Acute Toxicity	M Factor	Chronic Toxicity	M Factor	
L(E)C_50_ (mg/L)		NOEC (mg/L)	NRD Components	RD Components
0.1 < L(E)C_50_ ≤ 1	1	0.01 < NOEC ≤ 0.1	1	-
0.01 < L(E)C_50_ ≤ 0.1	10	0.001 < NOEC ≤ 0.01	10	1
0.001 < L(E)C_50_ ≤ 0.01	100	0.0001 < NOEC ≤ 0.001	100	10

L(E)C_50_—half maximal lethal/effective concentration; NOEC—no observed effect concentration; NRD—non-rapidly degradable; RD—rapidly degradable.

**Table 6:** Definition of M-factors for highly toxic components, including required parameters.

	LC_50_		EC_50_		EC_50_		NOEC		M_acute_	M_chronic_
	96 h (for Fish) (mg/L)	# Stud.	48 h (for Crustacean) (mg/L)	# Stud.	72 h or 96 h (for Algae) (mg/L)	# Stud.	Value	# Stud.		
As	**18.45**	2	-	-	-	-	0.456	4	1	1
Cd	0.111	34	0.124	37	**0.095**	1	0.017	71	10	10
Cr	≥10	11	**0.023**	3	≥5	3	0.0011	30	10	100
Cu	0.282	105	**0.086**	289	0.333	8	0.08	562	10	10
Hg	0.493	19	**0.0148**	4	0.175	2	0.05	1	10	10
Pb	1.088	8	3.054	4	**0.489**	1	0.335	36	1	1
Zn	1.02	43	0.813	40	**0.572**	1	0.194	65	1	1

**Table 7:** Calculations for hazard level definition.

#	Sum of Components Concentrations (%) Classified As:				
	Ac_1_ × M	Ch_1_ × M	(Ch_1_ × 10 × M) + Ch_2_	(Ch_1_ × 100 × M) + (Ch_2_ × 10) + Ch_3_	Ch_x_
4	6.20 × 10^−4^	5.12 × 10^−3^	5.12 × 10^−2^	5.12 × 10^−1^	9.35 × 10^−5^
5	5.63 × 10^−4^	5.06 × 10^−3^	5.06 × 10^−2^	5.06 × 10^−1^	8.26 × 10^−5^
7	6.04 × 10^−4^	5.10 × 10^−3^	5.10 × 10^−2^	5.10 × 10^−1^	8.87 × 10^−5^
18	3.49 × 10^−5^	1.07 × 10^−4^	1.07 × 10^−3^	1.07 × 10^−2^	2.00 × 10^−5^
19	4.36 × 10^−5^	8.86 × 10^−5^	8.87 × 10^−4^	8.87 × 10^−3^	2.66 × 10^−5^
20	3.27 × 10^−5^	1.50 × 10^−4^	1.50 × 10^−3^	1.50 × 10^−2^	2.59 × 10^−5^
21	4.33 × 10^−4^	3.01 × 10^−3^	3.01 × 10^−2^	3.01 × 10^−1^	4.96 × 10^−5^
22	9.41 × 10^−5^	5.26 × 10^−4^	5.26 × 10^−3^	5.26 × 10^−2^	1.36 × 10^−5^
23	1.85 × 10^−5^	6.35 × 10^−5^	6.37 × 10^−4^	6.38 × 10^−3^	2.42 × 10^−5^
24	4.10 × 10^−4^	2.87 × 10^−3^	2.87 × 10^−2^	2.87 × 10^−1^	4.75 × 10^−5^
25	2.11 × 10^−4^	1.67 × 10^−3^	1.67 × 10^−2^	1.67 × 10^−1^	2.95 × 10^−5^
29	1.77 × 10^−5^	4.47 × 10^−5^	4.49 × 10^−4^	4.49 × 10^−3^	1.23 × 10^−5^
30	5.28 × 10^−5^	1.16 × 10^−4^	1.16 × 10^−3^	1.16 × 10^−2^	2.07 × 10^−5^
48	1.93 × 10^−4^	9.94 × 10^−4^	9.94 × 10^−3^	9.94 × 10^−2^	2.82 × 10^−5^
49	2.60 × 10^−5^	1.08 × 10^−4^	1.08 × 10^−3^	1.08 × 10^−2^	7.69 × 10^−6^
50	2.31 × 10^−5^	9.78 × 10^−5^	9.78 × 10^−4^	9.79 × 10^−3^	6.96 × 10^−6^

Ac_1_—category acute 1, Ch_1_—category chronic 1, Ch_2_—category chronic 2, Ch_3_—category chronic 3, Ch_x_—all chronic categories.

## Appendix A

**Table A1:** Metal contents of RAs’ eluate (mg/kg).

#	As	Ba	Cd	Cr Total	Cu	Hg	Mo	Ni	Pb	Sb	Se	Zn	Chloride	Fluoride	Sulphate	DOC	TDS
1	<1.00 × 10^−3^	1.41	<d.l.	6.00 × 10^−1^	1.20 × 10^−1^		1.60 × 10^−1^	3.00 × 10^−2^	<5.00 × 10^−3^	1.00 × 10^−2^	7.40 × 10^−2^	5.00 × 10^−2^	1.71 × 10^2^	8.70	2.71 × 10^2^		
2	1.00 × 10^−2^	4.20 × 10^−1^	<d.l.	5.00 × 10^−1^	1.30 × 10^−1^		1.00 × 10^−1^	6.00 × 10^−2^	1.00 × 10^−2^	<1.00 × 10^−3^	7.00 × 10^−2^	1.40 × 10^−1^	3.21 × 10^2^	8.50	1.21 × 10^4^		
3	<1.00 × 10^−3^	3.30 × 10^−1^	<d.l.	4.50 × 10^−1^	3.00 × 10^−1^		2.70 × 10^−1^	4.00 × 10^−2^	<5.00 × 10^−3^	1.30 × 10^−2^	8.50 × 10^−2^	4.00 × 10^−2^	1.66 × 10^2^	8.70	1.09 × 10^3^		
4	1.00 × 10^−2^	2.80 × 10^−1^	<d.l.	5.00 × 10^−1^	1.00 × 10^−1^		1.70 × 10^−1^	5.00 × 10^−2^	2.00 × 10^−2^	1.00 × 10^−2^	7.50 × 10^−2^	1.70 × 10^−1^	5.00 × 10^1^	8.50	6.20 × 10^2^		
5	<1.00 × 10^−3^	2.00 × 10^−1^	<d.l.	5.00 × 10^−1^	5.00 × 10^−2^		8.00 × 10^−2^	4.00 × 10^−2^	1.00 × 10^−2^	2.00 × 10^−2^	8.50 × 10^−2^	1.20 × 10^−1^	8.06 × 10^1^	9.00	8.90 × 10^2^		
6	1.00 × 10^−2^	3.40 × 10^−1^	<d.l.	1.36	1.20 × 10^−1^		2.00 × 10^−1^	3.00 × 10^−2^	1.00 × 10^−2^	1.00 × 10^−2^	8.00 × 10^−2^	1.20 × 10^−1^	9.56 × 10^1^	9.20	8.00 × 10^2^		
7	1.00 × 10^−2^	8.60 × 10^−1^	<d.l.	5.00 × 10^−1^	9.00 × 10^−2^		1.60 × 10^−1^	6.00 × 10^−2^	1.00 × 10^−2^	1.00 × 10^−2^	8.70 × 10^−2^	1.20 × 10^−1^	7.00 × 10^1^	8.80	2.36 × 10^2^		
8	1.00 × 10^−2^	3.60 × 10^−1^	<d.l.	4.90 × 10^−1^	9.00 × 10^−2^	<5.00 × 10^−3^	8.00 × 10^−2^	1.00 × 10^−2^	<5.00 × 10^−3^	1.00 × 10^−2^	8.00 × 10^−2^	6.00 × 10^−2^	2.09 × 10^2^	9.00	6.45 × 10^3^		
9	1.00 × 10^−2^	2.80 × 10^−1^	<d.l.	4.80 × 10^−1^	7.00 × 10^−2^	1.00 × 10^−2^	1.00 × 10^−1^	1.00 × 10^−2^	<5.00 × 10^−3^	1.00 × 10^−2^	8.70 × 10^−2^	4.00 × 10^−2^	1.09 × 10^2^	8.50	1.45 × 10^3^		
10	1.00 × 10^−2^	2.90 × 10^−1^	<d.l.	1.10	1.10 × 10^−1^	<5.00 × 10^−3^	8.00 × 10^−2^	5.00 × 10^−2^	2.00 × 10^−2^	1.00 × 10^−2^	7.80 × 10^−2^	1.70 × 10^−1^	1.23 × 10^2^	8.00	2.27 × 10^3^		
11	1.00 × 10^−2^	1.20	<d.l.	5.20 × 10^−1^	1.10 × 10^−1^	<5.00 × 10^−3^	1.40 × 10^−1^	1.00 × 10^−1^	2.00 × 10^−2^	<1.00 × 10^−3^	7.00 × 10^−2^	1.40 × 10^−1^	5.00 × 10^1^	9.20	5.00 × 10^1^		
12	2.00 × 10^−2^	2.60 × 10^−1^	<d.l.	5.00 × 10^−1^	1.20 × 10^−1^	<5.00 × 10^−3^	6.00 × 10^−2^	5.00 × 10^−2^	2.00 × 10^−2^	1.00 × 10^−2^	8.00 × 10^−2^	1.30 × 10^−1^	5.00 × 10^1^	9.00	3.44 × 10^3^		
13	<1.00 × 10^−3^	1.70 × 10^−1^	<d.l.	7.40 × 10^−1^	1.00 × 10^−2^	7.00 × 10^−3^	7.00 × 10^−2^	1.00 × 10^−2^	<5.00 × 10^−3^	2.00 × 10^−2^	7.80 × 10^−2^	4.00 × 10^−2^	1.74 × 10^2^	8.90	3.45 × 10^3^		
14	2.00 × 10^−2^	3.50 × 10^−1^	<d.l.	4.50 × 10^−1^	2.10 × 10^−1^	8.00 × 10^−3^	2.20 × 10^−1^	5.00 × 10^−2^	2.00 × 10^−2^	2.00 × 10^−2^	8.00 × 10^−2^	1.60 × 10^−1^	5.00 × 10^1^	8.00	1.20 × 10^3^		
15	1.00 × 10^−2^	5.60 × 10^−1^	<d.l.	4.50 × 10^−1^	2.60 × 10^−1^	9.00 × 10^−3^	1.40 × 10^−1^	1.40 × 10^−1^	<5.00 × 10^−3^	1.00 × 10^−2^	7.80 × 10^−2^	5.00 × 10^−2^	7.80 × 10^1^	8.50	4.00 × 10^3^		
16	<1.00 × 10^−3^	5.40 × 10^−1^	<d.l.	5.60 × 10^−1^	2.00 × 10^−2^	<5.00 × 10^−3^	1.70 × 10^−1^	1.00 × 10^−2^	<5.00 × 10^−3^	<1.00 × 10^−3^	8.00 × 10^−2^	1.10 × 10^−1^	2.47 × 10^2^	8.00	5.75 × 10^3^		
17	<1.00 × 10^−3^	3.80 × 10^−1^	<d.l.	5.00 × 10^−1^	2.60 × 10^−1^	<5.00 × 10^−3^	1.50 × 10^−1^	5.00 × 10^−2^	3.00 × 10^−2^	1.00 × 10^−2^	9.00 × 10^−2^	2.60 × 10^−1^	1.04 × 10^2^	8.50	1.37 × 10^4^		
18	8.00 × 10^−3^	3.54 × 10^−1^	<d.l.	8.00 × 10^−3^	1.40 × 10^−2^	1.00 × 10^−3^	2.00 × 10^−3^	5.20 × 10^−2^	1.00 × 10^−3^	5.00 × 10^−3^	1.20 × 10^−3^	1.10 × 10^−1^	5.00 × 10^1^	<1.00	2.76 × 10^2^		
19	1.60 × 10^−2^	6.40 × 10^−2^	<d.l.	5.00 × 10^−3^	2.30 × 10^−2^	1.00 × 10^−3^	1.30 × 10^−2^	4.90 × 10^−2^	2.00 × 10^−3^	6.00 × 10^−3^	3.60 × 10^−2^	1.28 × 10^−1^	5.00 × 10^1^	<1.00	8.02 × 10^1^		
20	2.00 × 10^−3^	1.35 × 10^−1^	<d.l.	1.30 × 10^−2^	4.00 × 10^−3^	1.00 × 10^−3^	1.30 × 10^−2^	6.70 × 10^−2^	2.00 × 10^−3^	4.00 × 10^−3^	2.30 × 10^−2^	1.43 × 10^−1^	5.97 × 10^1^	<1.00	5.00 × 10^1^		
21	2.00 × 10^−3^	2.40		2.86 × 10^−1^	1.47 × 10^−1^		2.28 × 10^−1^	2.80 × 10^−2^		<d.l.	3.30 × 10^−2^	<d.l.					
22	1.00 × 10^−3^	2.19 × 10^−1^		4.80 × 10^−2^	4.50 × 10^−2^		2.70 × 10^−2^	7.00 × 10^−3^		2.30 × 10^−2^	2.00 × 10^−3^	1.00 × 10^−2^					
23	1.35 × 10^−1^	<d.l.		5.00 × 10^−3^	<d.l.		<d.l.	3.90 × 10^−2^		2.30 × 10^−2^	4.00 × 10^−2^	<d.l.					
24	1.30 × 10^−2^	5.90 × 10^−2^		2.73 × 10^−1^	1.36 × 10^−1^		9.40 × 10^−2^	1.00 × 10^−3^		3.60 × 10^−2^	1.60 × 10^−2^	<d.l.					
25	2.00 × 10^−3^	4.86 × 10^−1^		1.62 × 10^−1^	4.70 × 10^−2^		5.70 × 10^−2^	3.80 × 10^−2^		8.00 × 10^−3^	1.60 × 10^−2^	2.20 × 10^−2^					
26	2.00 × 10^−3^	4.37 × 10^−1^		7.06 × 10^−1^	1.06 × 10^−1^		8.20 × 10^−2^	2.70 × 10^−2^		1.10 × 10^−2^	7.00 × 10^−3^	1.00 × 10^−2^					
27	2.00 × 10^−3^	3.84 × 10^−1^		6.63 × 10^−1^	7.80 × 10^−2^		8.60 × 10^−2^	4.00 × 10^−2^		8.00 × 10^−3^	4.20 × 10^−2^	2.10 × 10^−2^					
28	3.00 × 10^−3^	1.17 × 10^−1^		1.02	<d.l.		4.20 × 10^−2^	7.00 × 10^−3^		7.00 × 10^−3^	4.20 × 10^−2^	<d.l.					
29	1.70 × 10^−2^	2.73 × 10^−1^		3.00 × 10^−3^	1.30 × 10^−2^		1.56 × 10^−1^	2.80 × 10^−2^		2.00 × 10^−2^	4.20 × 10^−2^	<d.l.					
30	8.00 × 10^−3^	3.36 × 10^−1^		7.00 × 10^−3^	4.50 × 10^−2^		1.28 × 10^−1^	3.50 × 10^−2^		4.80 × 10^−2^	6.40 × 10^−2^	<d.l.					
31	2.00 × 10^−3^	2.72 × 10^−1^		3.92	<d.l.		2.62 × 10^−1^	2.70 × 10^−2^		<d.l.	4.90 × 10^−2^	<d.l.					
32	5.00 × 10^−3^	2.13		1.02	2.33 × 10^−1^		3.81 × 10^−1^	1.36 × 10^−1^		<d.l.	3.80 × 10^−2^	<d.l.					
33	3.00 × 10^−3^	2.04		1.04	2.23 × 10^−1^		3.75 × 10^−1^	1.45 × 10^−1^		<d.l.	2.20 × 10^−2^	<d.l.					
34	6.00 × 10^−3^	2.05		9.57 × 10^−1^	2.28 × 10^−1^		4.06 × 10^−1^	1.42 × 10^−1^		<d.l.	4.00 × 10^−2^	<d.l.					
35	5.00 × 10^−3^	2.00		7.30 × 10^−1^	1.77 × 10^−1^		2.75 × 10^−1^	1.08 × 10^−1^		<d.l.	4.30 × 10^−2^	<d.l.					
36	4.00 × 10^−3^	9.28 × 10^−1^		7.82 × 10^−1^	1.40 × 10^−2^		9.80 × 10^−2^	3.10 × 10^−2^		<d.l.	8.00 × 10^−3^	<d.l.					
37	4.00 × 10^−3^	1.58		1.42	6.40 × 10^−2^		2.23 × 10^−1^	4.20 × 10^−2^		<d.l.	5.80 × 10^−2^	<d.l.					
38	9.20 × 10^−3^	3.16 × 10^−1^	<d.l.	2.62 × 10^−1^	3.19 × 10^−2^	<d.l.	7.75 × 10^−2^	3.26 × 10^−2^	<d.l.	5.00 × 10^−3^	<d.l.	<d.l.	6.38 × 10^1^	<2.00	1.12 × 10^4^		
39	5.30 × 10^−3^	2.21 × 10^−1^	<d.l.	5.48 × 10^−1^	2.79 × 10^−2^	<d.l.	5.44 × 10^−2^	9.80 × 10^−3^	<d.l.	3.40 × 10^−3^	<d.l.	<d.l.	5.47 × 10^1^	<2.00	4.15 × 10^3^		
40	<4 × 10^−1^	4.00	<1 × 10^−1^	<5 × 10^−1^	<1.00	<2 × 10^−1^	<3 × 10^−1^	<4 × 10^−1^	<5 × 10^−1^	<4 × 10^−1^	<2 × 10^−1^	<5 × 10^−1^	1.50 × 10^1^	<10.00	<30.00	8.80 × 10^1^	1,70 × 10^4^
41	<4 × 10^−1^	4.00	<1 × 10^−1^	<5 × 10^−1^	<1.00	<2 × 10^−1^	<3 × 10^−1^	<4 × 10^−1^	<5 × 10^−1^	<4 × 10^−1^	<2 × 10^−1^	<5 × 10^−1^	1.50 × 10^1^	<10.00	<30.00	8.80 × 10^1^	1,70 × 10^4^
42	<4 × 10^−1^	9.00	<1 × 10^−1^	5.00 × 10^−1^	<1.00	<2 × 10^−1^	6.00 × 10^−1^	<4 × 10^−1^	<5 × 10^−1^	<4 × 10^−1^	<2 × 10^−1^	<5 × 10^−1^	1.90 × 10^1^	<10.00	<30.00	1.10 × 10^1^	1,20 × 10^4^
43	<4 × 10^−1^	8.00	<1 × 10^−1^	7.00 × 10^−1^	<1.00	<2 × 10^−1^	1.00	<4 × 10^−1^	<5 × 10^−1^	<4 × 10^−1^	<2 × 10^−1^	<5 × 10^−1^	3.40 × 10^1^	<10.00	3.70 × 10^1^	1.00 × 10^2^	9,20 × 10^3^
44	5.21 × 10^−2^	2.16 × 10^−1^	0.00	6.51 × 10^−2^	5.77 × 10^−2^	0.00	3.06 × 10^−2^	5.50 × 10^−3^	0.00	1.45 × 10^−2^	4.10 × 10^−3^	2.36 × 10^−2^			2.56 × 10^3^		
45	1.35 × 10^−2^	3.80 × 10^−1^	0.00	1.42 × 10^−1^	2.83 × 10^−1^	0.00	1.41 × 10^−1^	2.12 × 10^−2^	0.00	2.37 × 10^−2^	1.77 × 10^−2^	5.83 × 10^−2^			8.23 × 10^3^		
46	1.05 × 10^−2^	1.93 × 10^−1^	0.00	9.10 × 10^−2^	0.00	0.00	5.47 × 10^−2^	6.20 × 10^−3^	5.30 × 10^−3^	1.75 × 10^−2^	1.10 × 10^−3^	3.11 × 10^−2^			9.68 × 10^3^		
47	2.58 × 10^−2^	3.14 × 10^−1^	0.00	5.40 × 10^−3^	1.62 × 10^−2^	0.00	7.92 × 10^−2^	2.37 × 10^−2^	0.00	1.80 × 10^−2^	2.18 × 10^−2^	6.97 × 10^−2^			1.22 × 10^4^		
48			<2.8 × 10^−4^	8.90 × 10^−2^	9.79 × 10^−2^			4.16 × 10^−2^	<0.0077			4.55 × 10^−2^	3.60 × 10^1^		4.88 × 10^2^	3.20 × 10^2^	
49			7.70 × 10^−4^	9.10 × 10^−3^	1.45 × 10^−2^			3.60 × 10^−2^	<0.0044			1.21 × 10^−2^	4.50 × 10^1^		7.03 × 10^2^	3.80 × 10^2^	
50			7.40 × 10^−4^	<8.3 × 10^−3^	1.32 × 10^−2^			3.84 × 10^−2^	<0.0044			4.60 × 10^−3^	6.20 × 10^1^		5.90 × 10^1^	2.50 × 10^2^	
51			6.30 × 10^−4^	3.00 × 10^−1^	6.67 × 10^−2^			2.86 × 10^−2^	<0.0044			1.11 × 10^−2^	1.85 × 10^2^		7.62 × 10^3^	8.50 × 10^1^	

<d.l.—bellow the detection limit.

## Appendix B

**Table A2:** Criteria for the application of the methodology (adapted from [34]).

Step	Description	Next Step
8	Confirm whether the material is just an inorganic product	8.1/8.2
8.1	Yes	9
8.2	No or not known	10
9	Check whether the chemical characterisation of the eluate of the material is available	9.1/9.2
9.1	Yes	19
9.2	No	17
10	Confirm whether the material is just an organic product	10.1/10.2
10.1	Yes	13
10.2	No or not known	11
11	Check whether the chemical characterisation of the eluate of the material is available	11.1/11.2
11.1	Yes	12
11.2	No	20
12	Check whether the obtained released content values are under the legal limit values (Table 3).	12.1/12.2
12.1	Yes	13
12.2	No	20
13	Check existing behaviour knowledge on degradability and the bioaccumulative potential	13.1/13.2
13.1	Yes	14
13.2	No	17
14	$\mathrm{Check}\;\mathrm{whether}\;\mathrm{the}\;\mathrm{material}\;\mathrm{is}\;\mathrm{rapidly}\;\mathrm{degradable}\;(e.g.,\;O_{2}{\mathrm{deplet}}_{28\;days}$ ≥ 0.7)	14.1/14.2
14.1	Yes	15
14.2	No	20
15	$\mathrm{Check}\;\mathrm{whether}\;\mathrm{the}\;\mathrm{material}\;\mathrm{can}\;\mathrm{bioaccumulate}\;(\mathrm{BCF}\;<\;500\;\mathrm{or}\;logK_{ow}<4$)	15.1/15.2
15.1	Yes	20
15.2	No	16
16	Output data: material may present an acceptable toxicity risk level	26 ^1^
17	Check whether the chemical composition of the material is available	17.1/17.2
17.1	Yes	19
17.2	No	18
18	Output data: insufficient data corresponding to x% (w/w)	26 ^1^
19	Check whether the obtained contents values are under the legal limit values (Table 3)	19.1/19.2
19.1	Yes	21
19.2	No	20
20	Output data: material may represent a toxicity concern level	33
21	Output data: material may present an acceptable toxicity risk level	26 ^1^
26	Check whether the material has any highly toxic component	26.1/6.2
27	Confirm whether the equation is true: ∑hazard ≥ 25%	27.1/27.2
27.1	Yes	31
27.2	No	28
28	Output data: mix with an acceptable toxicity level	33
29	Check whether the equation is true: ∑(Acute 1×M)≥25% (M according to Table 6)	29.1/29.2
29.1	Yes	31
29.2	No	30
30	Check whether any chronic option equation (see bottom of the figure and Section 3.3) is higher than 25%	30.1/30.2
30.1	Yes	31
30.2	No	32
31	Output data: final product may have a toxicity concern level	33
32	Output data: mix with acceptable toxicity level	35
33	End	

^1^ For this study in particular.

## Appendix C

**Table A3:** Methodology step results for individual RAs’ eluate.

#	8. Only Inorganic	10. Only Organic	11. Eluate CC	12.				13. Behav.	17. Comp.	18. Insuf. Data	19. ≤Limit?		20. Toxic	26. Highly Toxic	29.	30.	32.
				Metals and Anions ≤ Limit?		DOC ≤ Limit?									Ac_1_ × M ≥ 25%	Ch_opt._ ≥ 25%	RISK Ok
1	no	no	yes	no	Cr								risk				
2	no	no	yes	no	SO_4_^2−^								risk				
3	no	no	yes	no	SO_4_^2−^								risk				
4	no	no	yes	?	Hg?	?	DOC?, TDS?	no	no	100%				yes	no	no	ok
5	no	no	yes	?	Hg?	?	DOC?, TDS?	no	no	100%				yes	no	no	ok
6	no	no	yes	no	Cr								risk				
7	no	no	yes	?	Hg?	?	DOC?, TDS?	no	no	100%				yes	no	no	ok
8	no	no	yes	no	SO_4_^2−^								risk				
9	no	no	yes	no	SO_4_^2−^								risk				
10	no	no	yes	no	Cr, SO_4_^2−^								risk				
11	no	no	yes	no	Cr								risk				
12	no	no	yes	no	SO_4_^2−^								risk				
13	no	no	yes	no	Cr, SO_4_^2−^								risk				
14	no	no	yes	no	SO_4_^2−^								risk				
15	no	no	yes	no	SO_4_^2−^								risk				
16	no	no	yes	no	Cr, SO_4_^2−^								risk				
17	no	no	yes	no	SO_4_^2−^								risk				
18	no	no	yes	ok		?	DOC?, TDS?	no	no	100%				yes	no	no	ok
19	no	no	yes	ok		?	DOC?, TDS?	no	no	100%				yes	no	no	ok
20	no	no	yes	ok		?	DOC?, TDS?	no	no	100%				yes	no	no	ok
21	no	no	yes	?	Cd?, Hg?, Pb?, Cl^−^?, F^−^?, SO_4_^2−^?	?	DOC?, TDS?	no	no	100%				yes	no	no	ok
22	no	no	yes	?	Cd?, Hg?, Pb?, Cl^−^?, F^−^?, SO_4_^2−^?	?	DOC?, TDS?	no	no	100%				yes	no	no	ok
23	no	no	yes	?	Cd?, Hg?, Pb?, Cl^−^?, F^−^?, SO_4_^2−^?	?	DOC?, TDS?	no	no	100%				yes	no	no	ok
24	no	no	yes	?	Cd?, Hg?, Pb?, Cl^−^?, F^−^?, SO_4_^2−^?	?	DOC?, TDS?	no	no	100%				yes	no	no	ok
25	no	no	yes	?	Cd?, Hg?, Pb?, Cl^−^?, F^−^?, SO_4_^2−^?	?	DOC?, TDS?	no	no	100%				yes	no	no	ok
26	no	no	yes	no	Cr								risk				
27	no	no	yes	no	Cr								risk				
28	no	no	yes	no	Cr								risk				
29	no	no	yes	?	Cd?, Hg?, Pb?, Cl^−^?, F^−^?, SO_4_^2−^?	?	DOC?, TDS?	no	no	100%				yes	no	no	ok
30	no	no	yes	?	Cd?, Hg?, Pb?, Cl^−^?, F^−^?, SO_4_^2−^?	?	DOC?, TDS?	no	no	100%				yes	no	no	ok
31	no	no	yes	no	Cr								risk				
32	no	no	yes	no	Cr								risk				
33	no	no	yes	no	Cr								risk				
34	no	no	yes	no	Cr								risk				
35	no	no	yes	no	Cr								risk				
36	no	no	yes	no	Cr								risk				
37	no	no	yes	no	Cr								risk				
38	no	no	yes	no	SO_4_^2−^								risk				
39	no	no	yes	no	Cr, SO_4_^2−^								risk				
40	no	no	yes	?	Cd?, Hg?, Sb?, Se?	no	TDS						risk				
41	no	no	yes	?	Cd?, Hg?, Sb?, Se?	no	TDS						risk				
42	no	no	yes	no	Mo								risk				
43	no	no	yes	no	Cr, Mo								risk				
44	no	no	yes	no	SO_4_^2−^								risk				
45	no	no	yes	no	SO_4_^2−^								risk				
46	no	no	yes	no	SO_4_^2−^								risk				
47	no	no	yes	no	SO_4_^2−^								risk				
48	no	no	yes	?	As?, Ba?, Hg?, Mo?, Sb?, Se?, F^−^?	?	TDS?	no	no	100%				yes	no	no	ok
49	no	no	yes	?	As?, Ba?, Hg?, Mo?, Sb?, Se?, F^−^?	?	TDS?	no	no	100%				yes	no	no	ok
50	no	no	yes	?	As?, Ba?, Hg?, Mo?, Sb?, Se?, F^−^?	?	TDS?	no	no	100%				yes	no	no	ok
51	no	no	yes	no	SO_4_^2−^								risk				

?—no available information.
